# Gastrointestinal symptoms associated with PPE use among nurses in COVID-19 wards: a cross-sectional study

**DOI:** 10.3389/fpubh.2026.1827949

**Published:** 2026-07-27

**Authors:** Wenyu Li, Xutao Wu, Yani Yan, Zhiyang Xie, Tingting Huang, Lianlian Sun, Shaozhi Tian, Zhengjie Dai, Longcha Liu, Xiaoqun Xu

**Affiliations:** 1 Emergency Department, Longgang Branch, The First Affiliated Hospital of Wenzhou Medical University, Wenzhou, China; 2 Coronary Care Unit (CCU), The First Affiliated Hospital of Wenzhou Medical University, Wenzhou, China; 3 Department of Cardiovascular Medicine, Longgang Branch, The First Affiliated Hospital of Wenzhou Medical University, Wenzhou, China; 4 Nursing Department, The First Affiliated Hospital of Wenzhou Medical University, Wenzhou, China

**Keywords:** COVID-19, gastrointestinal symptoms, nausea, occupational health, personal protective equipment (PPE), psychological stress

## Abstract

**Objective:**

To investigate the prevalence of gastrointestinal symptoms (reflux, nausea, and vomiting) among nurses in COVID-19 isolation wards and identify associated factors, with a specific focus on discomfort caused by personal protective equipment (PPE).

**Study design:**

A cross-sectional survey.

**Methods:**

In March 2020, 354 of 368 eligible nurses (96.2% response rate) from the COVID-19 isolation wards of a designated hospital in Wenzhou, China, completed the survey. Data were collected using a demographic questionnaire, a self-rated PPE discomfort scale, the Gastroesophageal Reflux Disease Questionnaire (GerdQ), the Pittsburgh Sleep Quality Index (PSQI), and the Symptom Checklist-90 (SCL-90). Statistical assessments included univariate, correlation, and multivariable logistic regression analyses. Firth's penalized likelihood logistic regression and sensitivity analyses were applied to address sparse data in specific subgroups.

**Results:**

The overall prevalence of gastrointestinal symptoms in the cohort was 23.2% (82/354). Multivariable regression identified several independent factors significantly associated with these symptoms (all *P* < 0.01): severe PPE-induced discomfort (*OR* = 3.64, 95% *CI*: 1.95–6.80), elevated psychological stress (SCL-90 total score: *OR* = 1.14, 95% *CI*: 1.09–1.20), and poor sleep quality (PSQI total score: *OR* = 2.10, 95% *CI*: 1.51–2.92). Although univariate analyses suggested protective associations for prior intensive care unit (ICU) experience and male gender, these effects lost statistical significance following Firth correction. Temporally, 76.8% of symptomatic cases emerged within the first five days of the shift cycle.

**Conclusions:**

Gastrointestinal symptoms are prevalent among nurses in COVID-19 isolation wards and are strongly associated with PPE-induced discomfort, psychological stress, and sleep disturbances. Although the cross-sectional design precludes causal inference, these findings underscore an urgent need for the ergonomic optimization of PPE to alleviate heat stress and physical burden. Furthermore, implementing rapid adaptation training programs and integrating psychological and sleep support into routine occupational health surveillance are vital to safeguarding frontline clinicians.

## 1 Introduction

PPE is a critical barrier for frontline healthcare workers against highly contagious respiratory pathogens like SARS-CoV-2, MERS-CoV, and avian influenza (1, 2). Level 2 PPE—including N95 respirators, gowns, face shields, gloves, and shoe covers—is the standard base configuration in isolation wards (1). While effective at reducing exposure risk through physical isolation, a core infection control principle, the impermeable nature of PPE can cause heat stress, increased breathing resistance, fluid loss, and restricted mobility (3, 4). Although studies have focused on common PPE-related issues like headaches, dyspnea, and skin injuries (5–7), digestive symptoms such as nausea, reflux, and vomiting remain a largely overlooked yet critical concern. These symptoms pose significant and multifaceted risks. First, they directly impair physical and mental health, potentially exacerbating anxiety and leading to nutritional deficits and compromised immunity due to reduced food intake (8, 9). Second, vomiting can cause mask displacement, internal contamination, or even involuntary removal of PPE, substantially increasing exposure risk. The accumulation of vomit within a sealed mask may also lead to hypoxia or aspiration. Furthermore, managing a vomiting incident requires work interruption, ward exit, and PPE re-donning, which disrupts clinical continuity and jeopardizes patient safety. Beyond these immediate dangers, such events can instill psychological fear, undermining long-term morale and staff retention.

Notably, the clinical evidence base for PPE is generally weak. A COVID-19 era review highlighted a near-total lack of clinical studies on its effectiveness, fit, and tolerability (1). Furthermore, a Cochrane systematic review has graded the evidence in this field as generally low quality (10). Current literature lacks data on the specific physiological toll of PPE on the gastrointestinal system, particularly under the extreme conditions characteristic of the initial outbreak phase. This study provides a retrospective analysis of the “shock phase” of the pandemic (March 2020), capturing a unique window of high-intensity stress and prolonged PPE usage. This cross-sectional study was conducted to determine the prevalence of reflux, nausea, and vomiting among nurses in COVID-19 wards during this critical period. Although clinical protocols have since evolved, understanding the physiological limits of healthcare workers during such early-stage crises is essential for future pandemic preparedness.

## 2 Methods

### 2.1 Participants and timing

This investigation employed a cross-sectional survey design. The study was conducted in March 2020 at a designated COVID-19 hospital. A total census sampling method was employed, targeting the entire population of 368 nurses working in the isolation wards. The hospital administered a schedule of two consecutive weeks of clinical duty followed by a two-week centralized recovery period. To ensure accurate recall, the survey was distributed during the 1st week of this recovery phase, immediately after the nurses completed their 2-week clinical assignment. Ultimately, 354 nurses completed the survey, yielding a response rate of 96.2%.

Inclusion criteria were: (1) direct involvement in nursing COVID-19 patients in general or intensive care isolation units; (2) possession of a valid nursing license; and (3) provision of informed consent to participate voluntarily.

### 2.2 Survey instruments

Data were collected using a set of standardized instruments: a general information questionnaire, a self-developed item for assessing PPE discomfort, the Gastroesophageal Reflux Disease Questionnaire (GerdQ), the Pittsburgh Sleep Quality Index (PSQI), and the Symptom Checklist-90 (SCL-90).

#### 2.2.1 General information questionnaire

The general information questionnaire was researcher-designed. It covered demographics and work-related factors such as age, gender, work area, professional title, education level, and years of nursing experience.

#### 2.2.2 Assessment of PPE-induced discomfort

Given the lack of validated instruments specific to COVID-19 PPE usage at the onset of the pandemic, PPE-induced discomfort was evaluated using a self-developed single-item, 5-point scale. This item measured the overall subjective feeling of discomfort while wearing standard Level 2 PPE. The ratings were: 1 = None (almost no discomfort); 2 = Mild (slight discomfort, easily ignored); 3 = Moderate (noticeable discomfort, but tolerable); 4 = Severe (very uncomfortable, interfering with work); 5 = Extreme (extremely uncomfortable, unbearable).

#### 2.2.3 Assessment of reflux, nausea, and vomiting symptoms

Gastrointestinal symptoms were evaluated using the well-validated Chinese version of the Gastroesophageal Reflux Disease Questionnaire (GerdQ) (11) and a specific self-report item for vomiting. Although the standard GerdQ assesses the past 7 days, participants were explicitly instructed to evaluate their symptoms over the past 2 weeks to perfectly match their 14-day PPE exposure period in the isolation ward. The GerdQ consists of six items assessing the frequency of heartburn, regurgitation, epigastric pain, nausea, sleep disturbance, and medication use. Items are scored on a 0–3 Likert scale, yielding a total score of 0–18. In this study, the symptoms were categorized as follows:

- **Reflux (GERD)**: Defined as a GerdQ total score of ≥7. This threshold was selected based on screening guidelines to capture probable/mild reflux symptoms suitable for early occupational health surveillance, rather than strict clinical diagnosis.
- **Nausea:** Assessed independently using the score from GerdQ Item 4 (“How often did you have nausea?”).
- **Vomiting:** Assessed via a self-reported yes/no question regarding any episodes that occurred specifically while wearing PPE (i.e., intra-shift events) during the past 2 weeks of clinical duty.

These three symptoms were combined into a composite endpoint of ‘gastrointestinal symptoms' because, within the specific context of high-level PPE use, they share a critical occupational safety consequence: they all significantly increase the risk of protective seal compromise. Whether it is the intense discomfort of reflux, the physiological urge associated with nausea, or an actual vomiting episode, all three symptoms induce a strong, sometimes involuntary urge to adjust, loosen, or remove the N95 respirator and face shield to breathe or relieve pressure. Therefore, analyzing them as a composite outcome better captures the overall occupational hazard of PPE tolerance failure than evaluating them as purely independent clinical diseases.

#### 2.2.4 Assessment of symptom timing

For participants who reported any gastrointestinal symptoms (reflux, nausea, or vomiting), additional questions were asked to determine the timing of symptom onset. Specifically, participants were asked to identify the earliest or primary time of symptom occurrence using two separate items: (1) “Within which period of the two-week shift cycle did your symptoms first appear?” with response options of “First 5 working days,” “Middle period (days 6–10),” or “Last 5 working days (days 11–14)”; and (2) “Within which period of the workday did your symptoms first appear?” with response options of “Within the 1 h after donning PPE,” “During the middle hours of the shift,” or “Within the last hour of the shift.” Participants were instructed to select the single best answer for each item corresponding to the earliest onset of their symptoms. These timing items were presented only to nurses who reported at least one gastrointestinal symptom.

#### 2.2.5 Assessment of sleep quality

Sleep quality was evaluated using the Chinese version of the Pittsburgh Sleep Quality Index (PSQI) (12). The PSQI assesses seven components: subjective sleep quality, sleep latency, sleep duration, habitual sleep efficiency, sleep disturbances, use of sleeping medication, and daytime dysfunction. A global PSQI score greater than 8 is indicative of poor sleep quality. The scale demonstrated good internal consistency, with a Cronbach's α coefficient ranging from 0.82 to 0.83.

#### 2.2.6 Assessment of psychological status

Psychological status was measured with the Symptom Checklist-90 (SCL-90) (13). This 90-item self-report inventory covers ten symptom domains: somatization, obsessive-compulsive, interpersonal sensitivity, depression, anxiety, hostility, phobic anxiety, paranoid ideation, psychoticism, and additional items. Each item is rated on a 5-point scale of distress. The total score and factor scores were used as indicators of psychological stress.

### 2.3 Data collection

Data were collected using a web-based cross-sectional survey distributed via the Wenjuanxing platform (www.wjx.cn). The initial page of the survey detailed the study objectives, data confidentiality measures, and the voluntary nature of participation. Electronic informed consent was obtained by requiring participants to click an “I agree to participate” button before proceeding. This consent protocol received approval from the Ethics Committee of Clinical Research (ECCR) of The First Affiliated Hospital of Wenzhou Medical University [Approval No. 2020 (077)]. Subsequent sections presented the assessment instruments. To prevent duplicate submissions and ensure data integrity, the platform was configured to restrict responses to one per device. Furthermore, all research staff completed training in research ethics and survey methodology to guarantee standardized and ethically compliant data collection.

### 2.4 Statistical methods

Statistical analyses were performed using SPSS version 25.0 (IBM Corp., Armonk, NY, USA) and R version 4.2.0 (R Foundation for Statistical Computing, Vienna, Austria). Continuous variables are presented as means ± standard deviations (SDs) or medians (interquartile ranges, IQRs) depending on data distribution, and were compared using independent samples *t*-tests, Mann–Whitney U tests, or Kruskal–Wallis H tests, as appropriate. Categorical variables are expressed as frequencies and percentages, and were compared using Pearson's Chi-square tests or Fisher's exact tests.

Multivariable binary logistic regression was conducted to identify factors independently associated with gastrointestinal symptoms. Predictor variables were selected based on clinical plausibility and statistical significance in univariate analyses (*P* < 0.05). The final model incorporated gender, PPE-induced discomfort, prior ICU experience, SCL-90 total score, and PSQI total score. Model assumptions were rigorously tested: the Box-Tidwell transformation confirmed the linearity of continuous predictors (SCL-90 and PSQI) in the logit (*P* > 0.05), and variance inflation factors (VIFs) were calculated to rule out multicollinearity, utilizing an acceptable threshold of < 5. Model performance was evaluated using the Hosmer-Lemeshow goodness-of-fit test, Nagelkerke R^2^, and the area under the receiver operating characteristic curve (AUC).

Due to sparse event counts in specific subgroups (only three symptomatic cases each among male nurses and those with prior ICU experience), the standard maximum-likelihood logistic regression exhibited quasi-complete separation, yielding unstable odds ratios and excessively wide confidence intervals. Consequently, Firth's penalized likelihood logistic regression (via the logistf package in R) was employed as the primary multivariable analysis to derive robust estimates. A sensitivity analysis was also performed using a reduced model that excluded gender and ICU experience to verify the stability of the remaining predictors. Although the overall events per variable (EPV) ratio was acceptable (82 events / 5 variables = 16.4), both the Firth-corrected estimates and sensitivity findings are reported to transparently address the cell-sparsity issue. All tests were two-sided, and a *P*-value < 0.05 was considered statistically significant.

To address potential confounding between ICU experience and specific work area assignments, we analyzed their cross-distribution using Cramer's V and computed preliminary VIFs. A pilot model incorporating both variables revealed collinearity; therefore, work area was excluded from the final model. Subsequent VIFs for the final model remained below 2.0, confirming the absence of significant multicollinearity. Given the cross-sectional design, all reported relationships are associative rather than causal. Consequently, the findings are interpreted conservatively as hypothesis-generating rather than confirmatory.

## 3 Results

### 3.1 Univariate analysis of the prevalence of reflux, nausea, and vomiting

The overall prevalence of composite gastrointestinal symptoms in the cohort was 23.2% (82/354). When analyzed separately, the prevalence of probable reflux (GerdQ total score ≥7) was 15.3% (54/354), nausea (GerdQ item 4 score ≥1) was 19.8% (70/354), and acute intra-shift vomiting was 1.7% (6/354). Due to the high co-occurrence of these stress-induced symptoms, the majority of symptomatic nurses reported overlapping conditions.

Univariate analysis identified three factors significantly associated with symptom prevalence (all *P* < 0.05): work area, gender, and prior ICU experience (Table 1). Symptoms were significantly more prevalent among nurses in the critical care isolation unit (46.8%) compared with those in general isolation wards (16.6%), and among female nurses (25.6%) compared with males (6.7%). Notably, a marked disparity was observed regarding clinical background: nurses without prior ICU experience reported a symptom incidence of 29.9%, vs. only 3.3% among those with such experience.

**Table 1:** Demographic and work-related characteristics associated with composite gastrointestinal symptoms (reflux, nausea, and vomiting) (*N* = 354).

Variable	Gastrointestinal symptoms		Incidence (%)	χ^2^/t/Z	*P*
	**No (*n* = 272)**	**Yes (*n* = 82)**			
Work area					
Critical care isolation unit	41	36	46.8%	30.766	< 0.001
General Isolation Ward	231	46	16.6%		
Age^a^	29.0 (26.0–35.8)	29.0(26.0–36.0)		−0.229	0.819
Gender					
Male	42	3	6.7%	7.883	0.005
Female	230	79	25.6%		
Marital status^b^					
Married	167	43	20.5%	2.307	0.272
Unmarried	101	38	27.3%		
Divorced/Widowed	4	1	20.0%		
Only child					
No	236	68	22.4%	0.765	0.382
Yes	36	14	28.0%		
Technical title					
Nurse	51	18	26.1%	3.385	0.336
Senior Nurse	137	47	25.5%		
Nurse-in-Charge	73	14	16.1%		
Deputy Chief Nurse or above	11	3	21.4%		
Education level					
College or below	64	21	24.7%	0.439	0.803
Bachelor's Degree	207	61	22.8%		
Master's degree or above	1	0	0.0%		
Support nurses^b^					
Yes	12	3	20.0%	0.45	0.502
No	260	79	23.3%		
Infectious disease dept. experience					
No	242	77	24.1%	1.720	0.190
Yes	30	5	14.3%		
ICU work experience					
No	185	79	29.9%	26.664	< 0.001
Yes	87	3	3.3%		
Time of onset (Shift cycle)					
No Symptoms	272	-	-	332.868	< 0.001
First 5 Working Days	-	63	76.8%		
Middle Period	-	8	9.8%		
Last 5 Working Days	-	11	13.4%		
Time of onset (Single workday)					
No Occurrence	272	-	-	333.733	< 0.001
Within 1 h	-	55	67.1%		
Middle Hours	-	6	7.3%		
Within Last Hour	-	21	25.6%		
Occupational exposure experience					
No	258	76	22.8%	0.557	0.456
Yes	14	6	30.0%		
Previous PPE training					
No	31	8	20.5%	0.173	0.677
Yes	241	74	23.5%		

In this study, the critical care isolation unit was designated for severe and critical COVID-19 patients requiring advanced life support (e.g., high-flow oxygen therapy, mechanical ventilation, Continuous Renal Replacement Therapy [CRRT], or Extracorporeal Membrane Oxygenation [ECMO]). The general isolation unit admitted patients with mild or moderate symptoms, where nursing care primarily involved monitoring and basic support. “Support nurses” were defined as those deployed from other hospitals. A two-week shift cycle was implemented; the “Time of Onset (Shift Cycle)” is based on this schedule. The “Time of Onset (Single Workday)” refers to the 7 h period beginning when nurses started donning protective equipment. Composite Gastrointestinal Symptoms were defined as the presence of at least one of the following: reflux (GerdQ total score ≥7), nausea (GerdQ item 4 score ≥ 1), or self-reported intra-shift vomiting. ^a^indicates that the data did not follow a normal distribution. ^b^ indicates that Fisher's exact test was used.

Additionally, the number of symptomatic cases within specific subgroups was very low (male: *n* = 3; with ICU experience: *n* = 3). Such sparse events can cause quasi-complete separation in standard logistic regression, leading to unstable estimates. Therefore, we applied Firth's penalized likelihood correction in the multivariable analysis (see Table 4 and Statistical Methods) to obtain more reliable effect estimates. The apparent protective associations for these subgroups should be interpreted cautiously and require validation in larger cohorts.

Regarding the timing of symptom onset among the 82 symptomatic nurses, occurrences clustered early: 76.8% reported their first onset during the initial 5 days of the shift cycle, and 67.1% experienced their first episode within the 1 h of donning PPE.

Clinically, six nurses reported severe intra-shift vomiting episodes that necessitated emergency PPE doffing, resulting in potential occupational exposure and mandatory medical isolation for observation. Although the small sample size (*n* = 6) precluded a separate regression analysis for vomiting, we highlight these cases due to their profound occupational safety implications. All six cases occurred in female nurses working in the critical care isolation unit who lacked prior ICU experience. Furthermore, all reported extreme PPE discomfort and exhibited SCL-90 and PSQI scores above the cohort means. These preliminary descriptive findings suggest that vomiting events may serve as critical sentinel indicators of PPE tolerance failure and subsequent exposure risk.

Finally, regarding the cross-distribution of ICU experience and work areas, among the 77 nurses in the critical care isolation unit, 33 (42.9%) had prior ICU experience (30 asymptomatic and 3 symptomatic), compared with 57 (20.6%) among the 277 nurses in general isolation wards (all 57 were asymptomatic). The total number of nurses with prior ICU experience was 90 (87 asymptomatic and 3 symptomatic), consistent with the distribution reported in Table 1. The association between ICU experience and work area assignment was statistically significant (χ^2^ = 6.77, *P* = 0.009) but modest (Cramer's V = 0.14), confirming limited collinearity between the two variables.

### 3.2 Comparison of PPE discomfort, psychological status, and sleep quality stratified by symptom presence

As shown in Table 2, nurses reporting gastrointestinal symptoms experienced significantly greater discomfort from protective equipment (*P* < 0.001). The prevalence of symptoms rose sharply with the severity of reported discomfort, escalating from 8.3% in the “mild” group to 81.8% in the “extreme” group. Furthermore, this symptomatic group exhibited significantly higher total scores on both the SCL-90 and PSQI (*P* < 0.001), indicating elevated levels of psychological stress and poorer sleep quality compared to asymptomatic nurses.

**Table 2:** Comparison of PPE discomfort, psychological status, and sleep quality stratified by gastrointestinal symptoms (*N* = 354).

Variable	Gastrointestinal symptoms		Incidence (%)	χ^2^/t/Z	*P*
	**No (*n* = 272)**	**Yes (*n* = 82)**			
Discomfort from protective equipment					
None	21	5	19.2%	120.791	< 0.001
Mild	210	19	8.3%		
Moderate	31	20	39.2%		
Severe	6	20	76.9%		
Extreme	4	18	81.8%		
SCL-90 scores					
Somatization	1.19 ± 0.35	1.83 ± 0.57		9.63	< 0.001
Obsessive-compulsive	1.22 ± 0.29	1.35 ± 0.41		2.678	0.004
Interpersonal sensitivity	1.19 ± 0.23	1.26 ± 0.29		2.004	0.024
Depression	1.12 ± 0.25	1.26 ± 0.38		3.138	0.001
Anxiety	1.35 ± 0.41	1.73 ± 0.69		4.741	< 0.001
Hostility	0.61 ± 0.15	0.63 ± 0.12		1.244	0.108
Phobic anxiety	1.53 ± 0.49	1.75 ± 0.65		3.089	0.001
Paranoid ideation	0.31 ± 0.15	0.29 ± 0.13		1.177	0.121
Psychoticism	0.15 ± 0.03	0.16 ± 0.06		1.455	0.074
Other disorders	1.18 ± 0.29	1.35 ± 0.42		3.427	< 0.001
Total score	104.64 ± 21.69	142.49 ± 39.57		8.91	< 0.001
PSQI scores					
Sleep quality	1.91 ± 0.83	2.51 ± 0.75		6.191	< 0.001
Sleep latency	2.05 ± 0.57	2.39 ± 0.81		3.546	< 0.001
Sleep duration	1.97 ± 0.85	2.25 ± 0.92		2.458	< 0.001
Sleep efficiency	1.26 ± 0.47	1.41 ± 0.68		1.868	< 0.001
Sleep disturbance	1.97 ± 0.83	2.25 ± 0.86		2.605	< 0.001
Use of sleeping medication	1.61 ± 0.91	1.65 ± 0.97		3.32	< 0.001
Daytime dysfunction	1.78 ± 0.83	2.05 ± 0.92		2.381	< 0.001
Total score	8.63 ± 3.14	11.44 ± 3.41		6.72	< 0.001

### 3.3 Analysis of correlations between gastrointestinal symptoms and psychological/sleep factors

The results of the correlation analysis are presented in Table 3. Gastrointestinal symptoms showed significant correlations with several psychological and sleep measures. Specifically, they exhibited moderate positive correlations with somatization (*r* = 0.518, *P* < 0.001), anxiety (*r* = 0.271, *P* < 0.001), and sleep quality (*r* = 0.312, *P* < 0.001). Several other factors demonstrated weaker but statistically significant positive correlations (all *P* < 0.01), confirming the link between symptom severity and psychological distress.

**Table 3:** Correlation analysis between gastrointestinal symptoms and psychological/sleep factors (*N* = 354).

Variable	Gastrointestinal symptoms	
	**Correlation Coefficient (r)**	***P***
SCL-90 factors		
Somatization	0.518	< 0.001
Obsessive-compulsive	0.128	0.016
Anxiety	0.271	< 0.001
Phobic anxiety	0.185	< 0.001
PSQI factors		
Sleep quality	0.312	< 0.001
Sleep latency	0.167	0.002
Sleep duration	0.138	0.010
Sleep disturbance	0.152	0.004
Daytime dysfunction	0.105	0.049

### 3.4 Multivariate analysis of factors associated with gastrointestinal symptoms

As detailed in Table 4, the Firth-corrected multivariable logistic regression analysis identified PPE-induced discomfort, psychological stress, and sleep quality as independent factors significantly associated with gastrointestinal symptoms (all *P* < 0.01). Specifically, PPE-induced discomfort emerged as a potent factor, with each escalating level of discomfort significantly increasing the likelihood of symptoms (*OR* = 3.64, 95% *CI*: 1.95–6.80). Additionally, elevated psychological stress (*OR* = 1.14, 95% CI: 1.09–1.20 per one-point increase in SCL-90 total score) and poorer sleep quality (*OR* = 2.10, 95% *CI:* 1.51–2.92 per one-point increase in PSQI total score) were significant contributors.

**Table 4:** Multivariable logistic regression analysis of factors associated with gastrointestinal symptoms in nurses from COVID-19 isolation wards.

Variable	Firth-corrected OR (95% CI)	P	Sensitivity analysis (excluding gender & ICU) OR (95% CI)	*P*
Gender (male vs. female)	0.42 (0.15–1.12)	0.08	–	–
Discomfort from protective equipment (per level)	3.64 (1.95–6.80)	< 0.001	3.58 (1.92–6.67)	< 0.001
ICU work experience (yes vs. no)	0.31 (0.08–1.18)	0.09	–	–
SCL-90 total score (per point)	1.14 (1.09–1.20)	< 0.001	1.14 (1.09–1.19)	< 0.001
PSQI total score (per point)	2.10 (1.51–2.92)	< 0.001	2.11 (1.52–2.93)	< 0.001

Firth-corrected estimates address sparse-data bias. Sensitivity analysis excluded gender and ICU experience due to low event counts (only 3 symptomatic cases each), confirming the stability of the main effect estimates. The original unpenalized model (not shown) gave extreme ORs (0.023 and 0.003) for gender and ICU experience, which were likely artifacts.

Conversely, the Firth-corrected estimates for male gender and prior ICU experience were substantially attenuated and lost statistical significance (gender: adjusted *OR* = 0.42, 95% *CI*: 0.15–1.12, *P* = 0.08; ICU experience: adjusted *OR* = 0.31, 95% *CI*: 0.08–1.18, *P* = 0.09). This indicates that the apparent protective effects observed in the initial unpenalized model were likely artifacts driven by sparse data. Furthermore, a sensitivity analysis excluding these two predictors yielded virtually identical ORs for PPE discomfort (3.58), SCL-90 (1.14), and PSQI (2.11), thus confirming the robustness of these core associations.

Comprehensive model diagnostics supported the validity of these findings. The Box-Tidwell test confirmed the assumption of linearity in the logit for the continuous predictors (SCL-90: *P* = 0.31; PSQI: *P* = 0.27), and all variance inflation factors (VIFs) were below 2.0 (range: 1.12–1.87), ruling out significant multicollinearity. The model demonstrated good overall fit (Hosmer-Lemeshow χ^2^ = 8.34, df = 8, *P* = 0.40; overall model *P* < 0.001). It explained 34.2% of the variance (Nagelkerke *R*^2^ = 0.342) and exhibited strong discriminatory ability, with an AUC of 0.86 (95% CI: 0.82–0.90).

## 4 Discussion

### 4.1 Hazards and multifaceted risks of gastrointestinal symptoms in nurses from COVID-19 wards

This study analyzes data from March 2020, representing the initial “emergency response” phase of the COVID-19 pandemic. Although PPE protocols and supply chains have improved significantly since then, this dataset offers a valuable “maximum stress model.” It captures the physiological outcomes of healthcare professionals operating under conditions of profound uncertainty, high viral loads, and extended shifts—circumstances likely to recur during the early stages of future high-consequence infectious disease outbreaks.

Against this backdrop, our survey adds limited but important evidence regarding the prevalence of a relatively underreported symptom cluster (reflux, nausea, and vomiting) among nurses in COVID-19 isolation wards. Our detailed analysis reveals that the 23.2% overall prevalence predominantly reflects upper gastrointestinal distress—specifically nausea and probable GERD—rather than widespread severe acute events.

Nevertheless, the severe consequences observed in our cohort—specifically, instances where vomiting episodes necessitated emergency PPE doffing, increased the risk of occupational exposure, and led to mandatory medical isolation—provide critical evidence of a previously overlooked hazard. This substantial prevalence indicates a systemic occupational health issue rather than merely isolated incidents. Consequently, this work aligns with the call by Tsamakis et al. (14) to better understand the distinct health challenges faced by frontline clinicians, thereby filling a crucial knowledge gap by advancing from the mere awareness of a potential hazard to its empirical quantification and conceptual exploration.

### 4.2 Multifactorial analysis of associated factors

Beyond the high prevalence, this study identified a unique symptom timing: most onsets clustered in the first five days of the shift cycle (76.8%) and the 1 h of the workday (67.1%). This pattern corresponds to the peak stress response observed upon initial exposure to a new stressor, and is consistent with the COVID-19 ward environment as an associated factor. Subsequent multivariate analysis established that these symptoms are significantly associated with a confluence of psychological, environmental, equipment, and individual factors, highlighting a distinct occupational health profile specific to the pandemic context.

#### 4.2.1 The central role of psychological stress and sleep disturbance

Our results identify psychological stress and sleep disturbance as independent factors significantly associated with gastrointestinal symptoms. The markedly higher SCL-90 scores in the symptomatic group (142.49 ± 39.57 vs. 104.64 ± 21.69) reflect a heightened state of psychological distress, which is further corroborated by positive correlations with anxiety, phobic anxiety, and impaired sleep quality. These observations align with previous reports highlighting pervasive fear and anxiety among frontline nurses (15).

**Figure 1:**
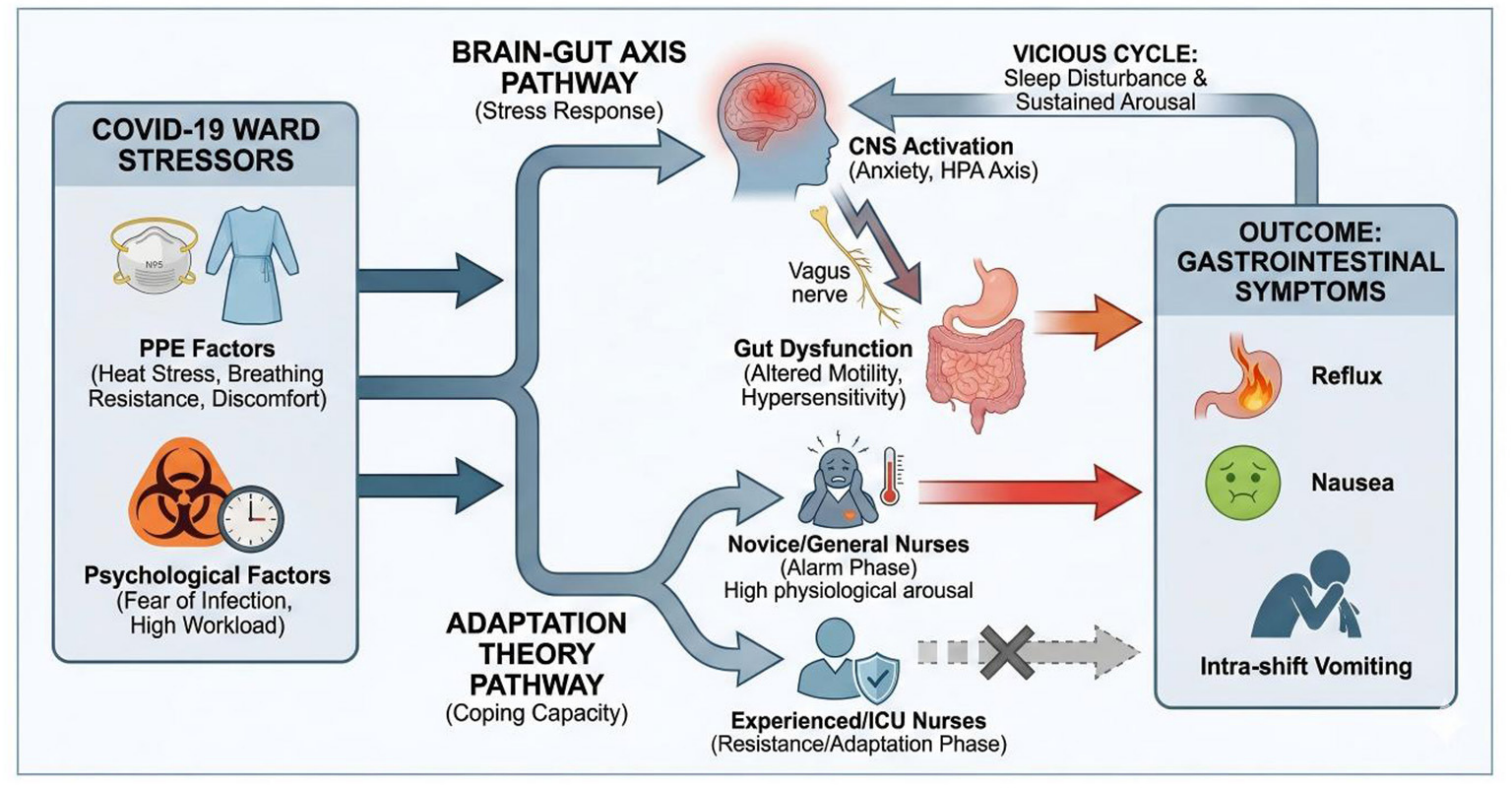
Proposed conceptual framework of PPE-associated gastrointestinal symptoms in nurses. This framework is presented as a hypothesis-generating model based on the observed cross-sectional associations, not as a demonstrated causal pathway.

To contextualize these findings, we propose a conceptual framework illustrating a hypothetical vicious cycle (Figure 1). In this model, severe work pressure and PPE-induced discomfort may induce anxiety, which can subsequently alter gastrointestinal function via the brain-gut axis (16, 17). Sleep disturbances may further exacerbate this GI distress through sympathetic nervous system activation (18); in turn, the GI symptoms themselves can disrupt sleep, potentially reinforcing the cycle. Consequently, PPE-related digestive issues may be embedded within a broader psychosomatic dysfunction. Importantly, this model remains speculative and warrants prospective validation, as the cross-sectional design of our study precludes the establishment of definitive temporal or causal relationships.

As detailed in the proposed multifactorial pathway (Figure 1): (A) Environmental stressors in the COVID-19 wards—notably PPE-induced heat stress and intense psychological pressure—may activate the central nervous system. (B) Through the brain-gut axis, this sustained stress can alter gut motility and visceral sensitivity, perpetuating the vicious cycle with sleep deprivation. (C) Framed within Stress Adaptation Theory, nurses equipped with prior ICU experience or robust coping mechanisms may successfully transition into the “Resistance Phase,” thereby buffering this physiological stress response. Conversely, novice nurses may remain in the “Alarm Phase,” resulting in a heightened physiological susceptibility to these psychosomatic symptoms.

#### 4.2.2 Discomfort from protective equipment: an overlooked strong associated factor

We identified PPE-related discomfort as a significant independent factor associated with gastrointestinal symptoms (OR = 3.64). A clear dose-response gradient was observed, with symptom incidence rising sharply from 8.3% in the “mild” group to 81.8% in the “extreme” group.

Mechanistically, the use of tight-fitting N95 respirators may increase intra-abdominal pressure during episodes of heightened respiratory effort, potentially promoting gastroesophageal reflux. Simultaneously, the hot and humid microclimate within the PPE ensemble contributes to heat stress (Figure 1), a known disruptor of gastrointestinal motility.

Psychophysiologically, persistent PPE-related discomfort functions as a chronic stressor, inducing sensory alterations that foster irritability and cumulative psychological pressure (19). This sustained stress is likely to activate both the hypothalamic-pituitary-adrenal (HPA) axis and the brain-gut axis, disrupting gut motility and visceral sensitivity, thereby triggering nausea and vomiting. Consequently, PPE-related discomfort should be viewed as a multisystem pathogenic factor, necessitating the implementation of comprehensive occupational health strategies (20).

#### 4.2.3 The protective effects of work experience and gender

The prevalence of gastrointestinal symptoms was approximately 2.8 times higher in the critical care isolation unit (46.8%) than in the general isolation ward (16.6%). This disparity aligns with Stress Adaptation Theory, which serves as a potential explanatory framework, although the cross-sectional design precludes causal inference. The convergence of intense stressors in the critical care unit maintains nurses in a prolonged state of high alert (the “alarm reaction” stage), elevating their physiological susceptibility to gastrointestinal dysfunction.

In univariate analyses, prior ICU work experience and male gender demonstrated suggestive associations with lower symptom prevalence (3.3% vs. 29.9% for ICU experience; 6.7% vs. 25.6% for males vs. females). However, after applying Firth's penalized likelihood correction to account for sparse event counts (only three symptomatic cases in each subgroup), these associations lost statistical significance (gender: *P* = 0.08; ICU experience: *P* = 0.09). This suggests that the apparent protective effects observed in the initial unpenalized model were likely inflated and should not be interpreted as robust evidence. Consequently, the limited number of cases in these demographic groups precludes definitive conclusions. Although experienced staff may possess superior coping strategies, our data cannot reliably quantify the magnitude of this protection. Future studies with larger and more balanced samples are required to clarify whether male gender and prior critical care training genuinely confer resilience.

Notably, ICU experience and work area were partially confounded, as nurses with prior ICU experience were more frequently assigned to critical care units. However, in a preliminary adjusted model, the effect of ICU experience remained significant while work area became non-significant. This suggests that the observed protective effect is better attributed to individual resilience and coping capacities acquired through prior critical care exposure, rather than the immediate work environment *per se*. This mediation effect also justifies the exclusion of work area from the final multivariable model.

### 4.3 Implications and future directions

In summary, gastrointestinal symptoms (reflux, nausea, and vomiting) among nurses in COVID-19 isolation wards appear to be associated with a complex interplay of occupational health factors. These findings underscore the critical need to systematically address the psychosocial and physiological risks faced by healthcare workers during public health emergencies, moving beyond a singular focus on infection control.

We propose an integrated intervention strategy to enhance preparedness for future public health emergencies:

**1. Preparedness-Oriented Ergonomic optimization:** The physiological burdens identified in this early-phase study highlight the need for advanced protective equipment in future strategic stockpiles. Healthcare systems and manufacturers should prioritize the development of “smart” PPE featuring improved breathability and active heat dissipation. This is crucial to minimize the physical discomfort and sensory deprivation that exacerbate stress during high-intensity outbreaks.

**2. Targeted Adaptation Training for Rapid Deployment:** Healthcare institutions should establish tiered training programs specifically designed for rapid response scenarios. These programs must emphasize rapid stress adaptation and physiological conditioning—particularly for nurses lacking prior critical care experience—to facilitate a smoother transition through the “alarm phase” during the onset of sudden public health crises.

**3. Holistic Support Systems:** Routine psychological screening and sleep management protocols must be incorporated into standard occupational health surveillance. Institutionalizing these measures is essential to safeguard the long-term wellbeing and occupational safety of frontline clinicians, ultimately ensuring a resilient workforce for future pandemics.

### 4.4 Limitations

This study has several limitations. First, the cross-sectional design precludes causal interpretation. We cannot determine whether psychological stress and sleep disturbances preceded gastrointestinal symptoms or vice versa. Furthermore, reverse causation is possible, as early gastrointestinal distress may have influenced nurses' retrospective ratings of PPE discomfort. Consequently, all reported associations are hypothesis-generating, and the proposed brain-gut axis model requires prospective validation.

Second, PPE-induced discomfort was assessed using a single-item, self-report scale to facilitate rapid data collection during the emergency response. This approach limits objective reliability and introduces potential common-method bias, as the score might capture overlapping constructs like general fatigue or respiratory burden. Similarly, minor domain overlap exists between standardized scales (e.g., sleep items in the GerdQ and PSQI), although this reflects the inherently interconnected psychosomatic nature of these symptoms.

Third, the timing data for symptom onset relied on retrospective recall of the earliest occurrence, rather than capturing the full temporal pattern (e.g., recurrent episodes) throughout the shift cycle. Future studies utilizing ecological momentary assessment could provide more granular temporal data.

Fourth, although we applied Firth's penalized likelihood regression to address sparse event counts in certain subgroups (e.g., male nurses and those with prior ICU experience, n = 3 each), the adjusted estimates maintained wide confidence intervals. Therefore, these potential protective effects remain exploratory and require validation in larger cohorts.

Finally, the single-center sampling and the specific March 2020 timeframe represent the initial “shock phase” of the pandemic. While this provides a valuable “maximum stress” model, it may limit generalizability to current clinical scenarios with evolved protocols. Future multi-center studies incorporating objective physiological measures (e.g., microclimate sensors, heart rate variability) are warranted to robustly confirm these findings.

## Data Availability

The raw data supporting the conclusions of this article will be made available by the authors, without undue reservation.
